# Primary Sjogren syndrome increases the risk of bisphosphonate-related osteonecrosis of the jaw

**DOI:** 10.1038/s41598-020-80622-5

**Published:** 2021-01-15

**Authors:** Pei-I Kuo, Tzu-Min Lin, Yu-Sheng Chang, Tsung-Yun Hou, Hui-Ching Hsu, Sheng-Hong Lin, Wei-Sheng Chen, Yi-Chun Lin, Li-Hsuan Wang, Chi-Ching Chang, Jin-Hua Chen

**Affiliations:** 1 Division of Rheumatology, Immunology and Allergy, Department of Internal Medicine, Yonghe Cardinal Tien Hospital, No. 80, Zhongxing St., Yonghe Dist., New Taipei City, 234 Taiwan; 2 Division of Rheumatology, Immunology and Allergy, Department of Internal Medicine, Taipei Medical University Hospital, Taipei, Taiwan; 3 Division of Allergy, Immunology, and Rheumatology, Department of Internal Medicine, Shuang Ho Hospital, Taipei Medical University, New Taipei City, Taiwan; 4 Division of Rheumatology/Immunology/Allergy, Department of Internal Medicine, Wan Fang Hospital, Taipei Medical University, Taipei, Taiwan; 5 Division of Rheumatology/Immunology/Allergy, Department of Internal Medicine, Taipei Veterans General Hospital, Taipei, Taiwan; 6 Division of Allergy, Immunology and Rheumatology, Department of Internal Medicine, School of Medicine, College of Medicine, Taipei Medical University, Taipei, Taiwan; 7 Biostatistics Center, College of Management, Taipei Medical University, Taipei, Taiwan; 8 School of Pharmacy, College of Pharmacy, Taipei Medical University, Taipei, Taiwan; 9 Department of Pharmacy, Taipei Medical University Hospital, Taipei, Taiwan; 10 Graduate Institute of Data Science, College of Management, Taipei Medical University, Taipei, Taiwan

**Keywords:** Drug discovery, Genetics, Rheumatology, Risk factors

## Abstract

The risk of bisphosphonate-related osteonecrosis of the jaw (BRONJ) in primary Sjogren syndrome (pSS) has rarely been explored. To explore the association between BRONJ and pSS, we conducted a population-based propensity-score-matched cohort study using Taiwan’s National Health Insurance Research Database, including pSS patients receiving antiosteoporotic therapy and patients without pSS receiving antiosteoporotic therapy. A 1:4 matched-pair cohort based on propensity score was created. The stratified Cox proportional hazards model compared the risk of BRONJ in the pSS and non-pSS groups. In the study, 23,280 pSS patients and 28,712,152 controls were enrolled. After matching, 348 patients with pSS receiving antiosteoporotic drugs and 50,145 without pSS receiving antiosteoporotic drugs were included for analysis. The risk of developing BRONJ was 1.96 times higher in pSS patients compared with non-pSS patients after adjustment for age, sex, and comorbidities. No dose–response effect was observed in the bisphosphonate-treated pSS cohorts, documented as the cumulative defined daily doses of either < 224 or ≥ 224 (hazard ratio [HR]: 2.407, 95% confidence interval [CI] 1.412–7.790; HR: 2.143, 95% CI 1.046–4.393, respectively) increased risk of developing osteonecrosis of the jaw. In conclusion, the risk of BRONJ is significantly higher in patients with pSS compared with the general population.

## Introduction

Osteoporosis, characterized by a systemic impairment of bone mass, strength, and microarchitecture that results in fragility fracture^1^, is a major health concern and burden. It may affect the quality of life and cost effective. Therefore, prevention and treatment of oxsteoporosis become an important issue. There are many available medications for the treatrment of osteoporosis, such as BPs, monoclonal antibody medications, hormone related therapy and bone building medication. BPs has been used for long time. Bisphosphonates (BPs) are widely used for the treatment of osteoporosis. BP-related osteonecrosis of the jaw (BRONJ) is a rare but serious complication that limits BP use. However, our understanding of the association between BPs and BRONJ is limited. Genetic associations have recently been identified. BP modulation of the gene expression involved in osteoblast function may have possible implications for patients with BRONJ^2^. Nicoletti et al. found that RNA-binding motif single stranded interacting protein 3 (RBMS3) is a genome-wide pharmacogenetic in BRONJ^3^.

Primary Sjogren syndrome (pSS) is a chronic autoimmune disease. The worldwide prevalence of pSS is estimated between 0.05% and 4.8%, and the female-to-male ratio is 9:1^4^. pSS is characterized by focal mononuclear cell infiltration of the salivary and lacrimal glands^5^. The commonest symptoms of the disease are dry eyes or dry mouth (sometimes both together) and feeling tired and achy. The glands are destructed gradually and the process is usually irreversible. The pathogenesis of this disease is obscure. Cellular pathology has recently been discovered. Research on the crucial immune as well as nonimmune roles of salivary gland epithelial cells has added new dimensions to the understanding of its pathogenesis. Chronic pSS patients have poor quality of life and spend much medical sources.

Now a day, medical advancement brough more unknown into known. Genetic study helps a lot. One study demonstrated that RBMS3 is a novel susceptibility gene that predisposes women to pSS, potentially through modulating acinar apoptosis and transforming growth factor-beta (TGF-β) signaling in the targeted exocrine system^4^. Since RBMS3 is also a genome-wide pharmacogenetic in BRONJ and RBMS3 is also present in pSS patient, there may be a relationship between pSS and BRONJ. Since there is no studies to prove or identify the relationship between BRONJ and pSS cases, therefore, we hypothesized that patients with pSS are predisposed to the development of BRONJ. To verify this hypothesis, we utilized a nationwide population-based registry, Taiwan National Health Insurance Research Database (NHIRD), for further investigation on the association between BRONJ and pSS.

## Results

### Baseline characteristics of the study population

**Figure 1:**
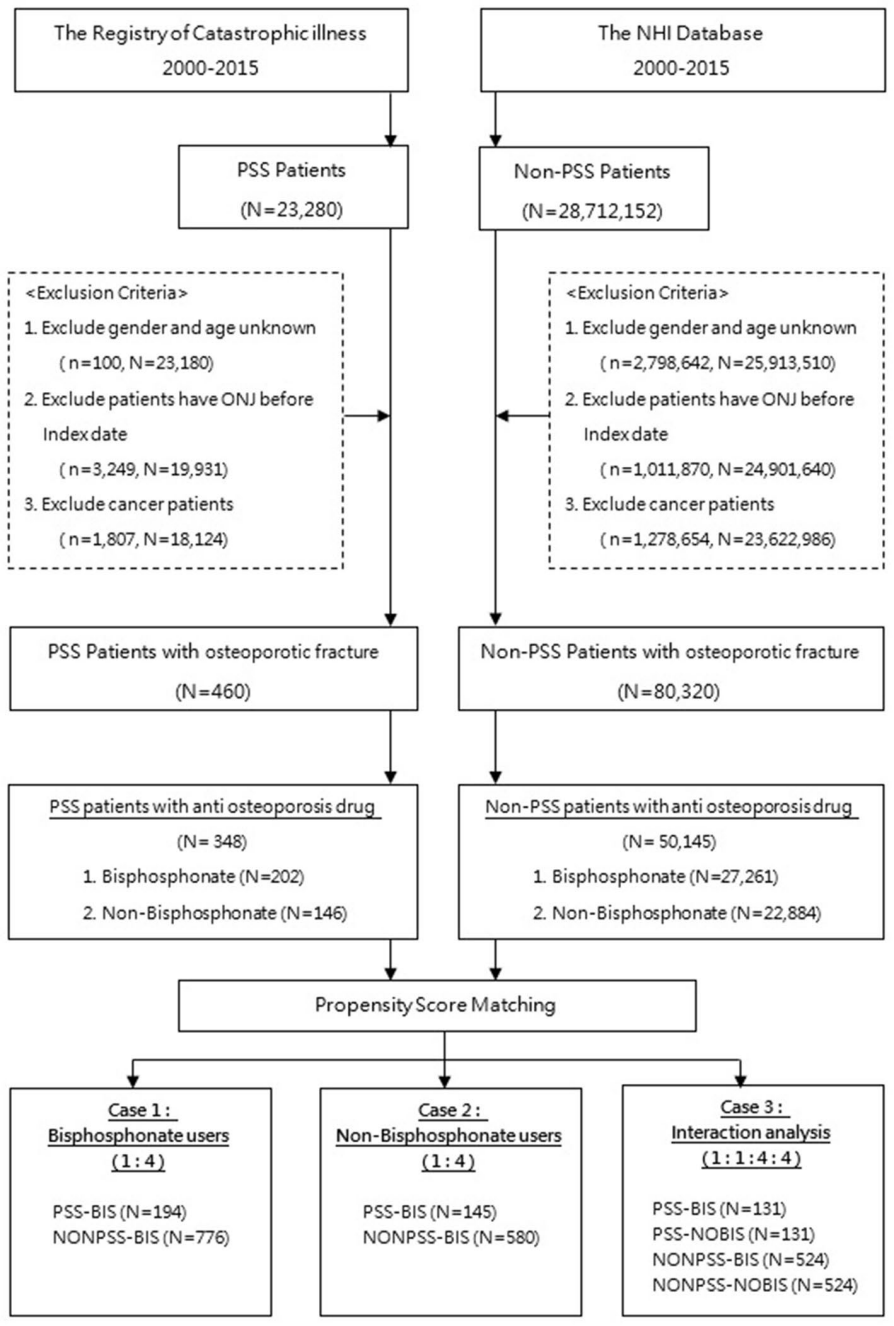
Flow chart of the study design.

Figure 1 depicted the study flowchart. A total of 23,280 patients with pSS and 28,712,152 patients without were identified for years 2000–2015. After excluding patients according to the criteria aforementioned, 50,493 patients were included in the study with 348 subjects in the pSS group and 50,145 subjects in the non-pSS group. Following propensity score matching, 1,695 patients were matched in each group for the final analysis.

Table 1 presents the baseline characteristics of BP users and their matched controls. A total of 348 patients with pSS and osteoporotic fractures were identified, of whom female was more prevalent (95.88%). The mean age at the time of diagnosis of pSS and osteoporotic fracture in the BP group was 70.81 years (standard deviation [SD] 9.79). The mean age at the time of diagnosis of pSS and osteoporotic fracture in the non-BP group was 71.30 years (SD 8.77). The comorbidities between the BP and non-BP groups are almost identical. The mean follow-up duration for each group was as follows: pSS with BPs, 6.69 ± 3.86 years; non-pSS with BPs, 7.26 ± 4.05 years; pSS without BP use, 7.42 ± 4.14 years; and non-pSS without BP use, 7.59 ± 4.15 years. Patients with pSS using BP had a significantly higher prevalence of ONJ than the non-pSS group did (9.79% vs 5.67%, p = 0.0371; Table 1).

**Table 1:** Baseline characteristics of bisphosphonate and non-bisphosphonate users in pSS or non-pSS patients.

	Bisphosphonate user			Non-Bisphosphonate user		
	Case (pSS patients) (N = 194)	Comparison (non-pSS patients) (N = 776)		Case (pSS patients) (N = 145)	Comparison (non-pSS patients) (N = 580)	
	N (%)	N (%)	*P* value	N (%)	N (%)	*P* value
Gender			0.6669			1.0000
Female	186 (95.88%)	749 (96.52%)		142 (97.93%)	568 (97.93%)	
Male	8 (4.12%)	27 (3.48%)		3 (2.07%)	12 (2.07%)	
Age			0.8007			0.9823
≤ 50	7 (3.61%)	21 (2.71%)		3 (2.07%)	12 (2.07%)	
51–60	19 (9.79%)	67 (8.63%)		11 (7.59%)	47 (8.10%)	
61–70	58 (29.90%)	238 (30.67%)		48 (33.10%)	188 (32.41%)	
71–80	79 (40.72%)	348 (44.85%)		65 (44.83%)	249 (42.93%)	
≥ 81	31 (15.98%)	102 (13.14%)		18 (12.41%)	84 (14.48%)	
Mean (SD)	70.81 (9.79)	71.24 (9.36)	0.5741	71.30 (8.77)	71.62 (8.91)	0.6959
Median (IQR)	72 (13)	72 (11)	0.6754	72 (11)	72 (12)	0.6505
Comorbidities						
Diabetes mellitus	30 (15.46%)	121 (15.59%)	0.9647	31 (21.38%)	126 (21.72%)	0.9282
Dyslipidemia	49 (25.26%)	194 (25.00%)	0.9409	24 (16.55%)	89 (15.34%)	0.7201
Hypertension	107 (55.15%)	433 (55.80%)	0.8716	77 (53.10%)	327 (56.38%)	0.4775
Hypothyroidism	3 (1.55%)	11 (1.42%)	0.8929	5 (3.45%)	13 (2.24%)	0.4035
Hyperthyroidism	7 (3.61%)	33 (4.25%)	0.6864	4 (2.76%)	19 (3.28%)	0.7506
Anemia	19 (9.79%)	65 (8.38%)	0.5301	21 (14.48%)	92 (15.86%)	0.6821
Chronic kidney disease	6 (3.09%)	22 (2.84%)	0.8479	7 (4.83%)	25 (4.31%)	0.7862
Esophagitis or ulcers	23 (11.86%)	92 (11.86%)	1.0000	10 (6.90%)	40 (6.90%)	1.0000
Peptic ulcer	71 (36.60%)	290 (37.37%)	0.8421	55 (37.93%)	223 (38.45%)	0.9088
Follow-up Time (days)						
Mean (SD)	2443 (1410)	2650 (1479)	0.0789	2707 (1511)	2771 (1516)	0.6488
Median (IQR)	2341 (2049)	2523 (2292)	0.0893	2534 (2736)	2665 (2336)	0.6077
ONJ			0.0371			0.0971
No	175 (90.21%)	732 (94.33%)		140 (96.55%)	538 (92.76%)	
Yes	19 (9.79%)	44 (5.67%)		5 (3.45%)	42 (7.24%)	

*pSS* primary Sjogren syndrome, *ONJ* osteonecrosis of the jaw.

### Risk of osteonecrosis of the jaw in bisphosphonate and non-bisphosphonate groups among patients with and without primary Sjogren syndrome

Table 2 illustrated the correlation between BP exposure and ONJ development among pSS and non-pSS patients. pSS patients using BPs had a significantly higher risk of ONJ (Adjusted HR: 1.96, 95% CI 1.14–3.38) than the non-pSS group using BPs. No increased risk of ONJ was observed among patients with pSS and the non-pSS cohort without BP exposure.

**Table 2:** Analysis of the Cox Proportional Hazard Model with cluster data for bisphosphonate and non-bisphosphonate users in pSS cohorts.

Case 1: bisphosphonate users						
pSS	Event	Incidence rate	IRR	95 CI for IRR	Adj. HR^‡^	95 CI for Adj. HR
No (Ref.)	44	1071.30	Ref		Ref	
Yes	19	2133.49	1.99*	(1.16–3.41)	1.96*	(1.14–3.38)

Case 2: non-bisphosphonate users						
pSS	Event	Incidence rate	IRR	95 CI for IRR	Adj. HR^‡^	95 CI for Adj. HR
No (Ref.)	42	1443.11	Ref		Ref	
Yes	5	473.50	0.52	(0.20–1.30)	0.53	(0.22–1.28)

(1) *0.01 ≤ *p* < 0.05, **0.0001 ≤ *p* < 0.01, ***p < 0.0001. (2) ^‡^ADJ. HR was adjusted by sex, comorbidities, and age at the initiation of bisphosphonate use.

*pSS* primary Sjogren syndrome, *IRR* incidence rate ratio, *95 CI* 95% confidence interval, *Adj. HR* adjusted hazard ratio, *Ref* reference.

Regarding the amount of BPs stratified by cDDD, no dose-dependent effect was observed in the BP subgroups for both the pSS and non-pSS cohorts. For patients with pSS and BP exposure, both the cDDD of < 224 and cDDD of ≥ 224 increased the risk of ONJ (HR: 2.407, 95% CI 1.412–7.790; HR: 2.143, 95% CI 1.046–4.393, respectively). No increased risk of ONJ with non-BP exposure stratified by cDDD was noted (Table 3).

**Table 3:** Stratified analysis of the cDDD for bisphosphonate and non-bisphosphonate users.

Case 1: bisphosphonate users				
pSS	Bisphosphonate users cDDD < 224		Bisphosphonate users cDDD ≥ 224	
	ADJ. HR	95% CI	ADJ. HR	95% CI
No (Ref.)	1.000		1.000	
Yes	2.407*	(1.412–7.790)	2.143*	(1.046–4.393)

Case 2: Non-bisphosphonate users				
pSS	Non-bisphosphonate users cDDD < 224		Non-bisphosphonate users cDDD ≥ 224	
	ADJ. HR	95% CI	ADJ. HR	95% CI
No (ref.)	1.000		1.000	
Yes	0.447	(0.128–1.562)	0.549	(0.121–2.493)

(1) *0.01 ≤ *p* < 0.05, **0.0001 ≤ *p* < 0.01, ***p < 0.0001. (2) ^‡^ADJ. HR was adjusted by sex, comorbidities, and age at the initiation of bisphosphonate use.

*cDDD* cumulative dose with defined daily dose, *95% CI* 95% confidence interval, *ADJ. HR* adjusted hazard ratio, *Ref* reference.

### Interaction analysis between primary Sjogren syndrome and antiosteoporotic drug use

Overall, only the pSS with BP group had the highest risk of BRONJ (Adjusted HR: 3.01, 95% CI 1.50–6.06; Table 4). The 3 other groups did not show an increased risk of ONJ.

**Table 4:** Interaction analysis between pSS and anti-osteoporosis drugs users in Case 3.

Group	Event	Incidence Rate	IRR	95 CI for IRR	Adj. HR^‡^	95 CI for Adj. HR
pSS-BIS	14	2327.95	3.05**	(1.56–5.97)	3.01**	(1.50–6.06)
pSS-NONBIS	4	615.90	0.81	(0.28–2.34)	0.79	(0.29–2.20)
NONpSS-BIS	29	1051.65	1.38	(0.79–2.40)	1.34	(0.77–2.34)
NONpSS-NONBIS (Ref.)	22	762.55				

(1) *0.01 ≤ *p* < 0.05, **0.0001 ≤ *p* < 0.01, ***p < 0.0001. (2) ^‡^ADJ. HR was adjusted by sex, comorbidities, and age at the initiation of bisphosphonate use.

*pSS* primary Sjogren syndrome, *IRR* incidence rate ratio, *95 CI* 95% confidence interval, *Adj. HR* adjusted hazard ratio, *pSS-BIS* primary Sjogren syndrome with bisphosphonate user, *pSS-NONBIS* primary Sjogren syndrome with non-bisphosphonate user, *NONpSS-BIS* non-primary Sjogren syndrome with bisphosphonate user, *NONpSS-NONBIS* non-primary Sjogren syndrome with non-bisphosphonate user, *Ref* reference.

### Cumulative incidence of osteonecrosis of the jaw with bisphosphonate exposure in the primary Sjogren syndrome and non-primary Sjogren syndrome cohorts

**Figure 2:**
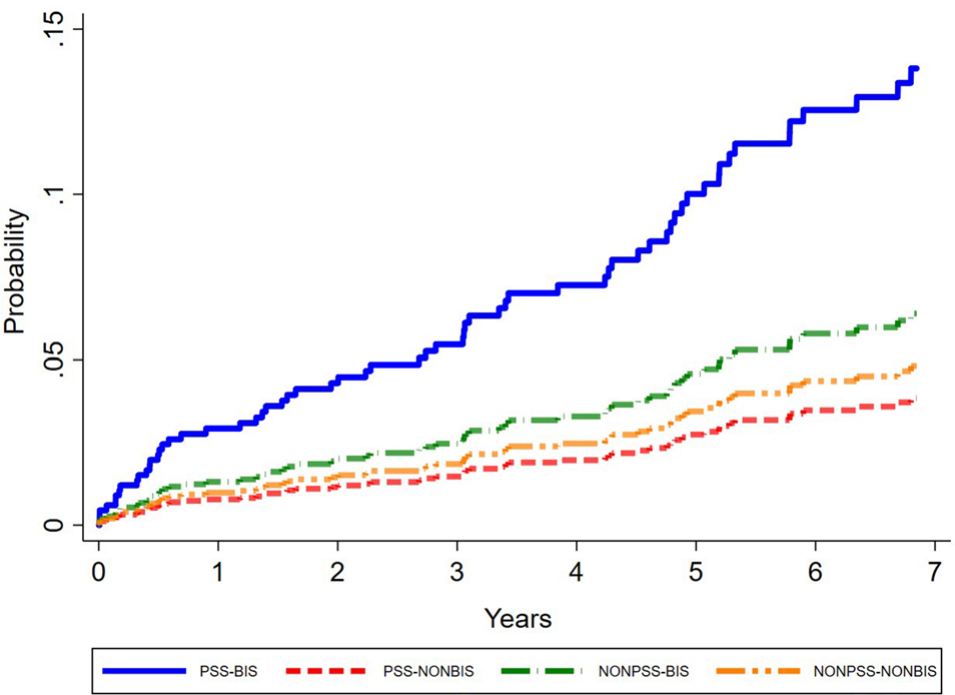
Cumulative incidence of ONJ predicted by Cox model in terms of bisphosphonate and non-bisphosphonate exposure among pSS and non-pSS patients.

A Kaplan–Meier analysis revealed the cumulative incidence of ONJ development in those BP exposures and non-BP exposures in the pSS and non-pSS cohorts (Fig. 2). The cumulative incidence of ONJ in the pSS patients with BP exposure was significantly higher than in the matched control (*p* = 0.0024).

## Discussion

According to our review of the relevant literature, this is the first nationwide population-based study to investigate the relationship between pSS and the risk of BRONJ. The results indicated that patients with pSS using BPs had an approximately two-fold higher risk of BRONJ as compared with a non-pSS cohort. Therefore, we postulated that pSS increased the risk of BRONJ.

Osteoporosis is a key concern in menopausal women. The main treatment options for osteoporosis include BP and non-BP medication. One of the most serious adverse effects of using BPs is BRONJ, especially among cancer patients^6,7^. Although it is rare^8^, the consequences of BRONJ may be irreversible. Several studies have sought to determine the risk factors of BRONJ. In oral surgical procedures, tooth extractions and infection in jaw bone were considered the main risk factors for developing ONJ while receiving antiresorptive therapy^9^. The evidence-based mechanisms of ONJ pathogenesis included disturbed bone remodeling, inflammation or infection, altered immunity, soft tissue toxicity, and angiogenesis inhibition. The role of dental infections and the oral microbiome was central to ONJ, and the systemic conditions such as rheumatoid arthritis^10^ and diabetes mellitus contributed through their effects on immune resiliency^11^. Studies have investigated the relationship between genetics and BP-induced BRONJ^12–14^. One study uncovered a significant increase in TFG-β1, TGF-βR1, TGF-βR2, TGF-βR3, and vascular endothelial growth factor (VEGF) expression and a significant reduction in RUNX-2, Col-1, OSX, OSC, BMP-2, BMP-7, ALP, and RANKL expression, whereas osteoprotegerin (OPG) expression varied according to the dose and cell line^2^. These findings suggested that osteoblasts may play an important role in BRONJ development. In 2012, Nicoletti et al. published a report in *The Oncologist* suggesting that RBMS3 has a pivotal role in BRONJ etiology^3^. RBMS3 is a binding protein for Prx 1, a homeobox transcriptional factor that upregulates collagen type I in fibroblasts^15^. Variations in RBMS3 (rs 10,510,628) and COLIA (rs 180,001) had previously been associated with a decrease in bone mass and subsequent osteoporotic fractures, linking both genes with bone turnover^16,17^. In terms of possible etiopathogenic mechanisms, it was assumed that ONJ could be caused by BP-associated suppressed bone turnover that led to decreased blood flow, bone cell necrosis, and apoptosis^18^. BPs also downregulated collagen type I synthesis in human gingival fibroblasts and osteoblasts^19^. In 2016, a genetic study determined that GTF21 (rs 117,026,326) and RBMS3 (rs 13,079,920, and rs 13,072,846) were significant susceptibility genes that were associated with pSS in women^4^. RBMS3 was highly expressed in the salivary gland^20^ and was demonstrated to inhibit cell proliferation and induce apoptosis^21^. Therefore, the RBMS3 gene might increase the risk of BRONJ in patients with pSS using BPs.

In our study, we observed that usage of BPs increased the risk of BRONJ in patients with pSS, while BPs dosage was not relevant. Other studies had demonstrated that the incidence of BRONJ increased with the duration of BP therapy^22–24^, in which our study also supported this finding (Fig. 2). Many studies had further identified an increased risk of BRONJ after the administration of BPs for more than 3 years^25,26^.

The strengths of the present study included large sample size, a large validation cohort, and a long-term ascertainment of medication information. However, the present study had limitations. First, although the NHI Bureau routinely and randomly checked patient charts to ensure the quality of claims from medical institutions, the possibility of miscoding or misclassification could not be completely ruled out. However, such confounder would apply to both the pSS and control cohorts, and therefore the present findings are expected to underestimate, rather than overestimate, the magnitude of the association between pSS and BRONJ. Second, the relationship between disease activity and the severity of pSS could not be analyzed. Finally, information regarding laboratory and clinical data was not readily available in the administrative database. In the future, further prospective studies will be expected to confirm whether the activity and severity of pSS or clinical biomarkers increase the risk of BRONJ.

In conclusion, this nationwide long-term retrospective cohort study demonstrated that BRONJ risk was significantly higher in patients with pSS treated with BPs compared with the general population. The mechanism of BRONJ in patients with pSS needs to be further studied in the future.

## Materials and methods

### Data source

The medical data were obtained from the National Health Research Institute (NHRI). The National Health Insurance (NHI) program was initiated in 1995 to provide healthcare for citizens and residents of Taiwan. Enrolment in this program is mandatory, resulting in a coverage rate of nearly 99%^27^. The Taiwan National Health Insurance Research Database (NHIRD), which is maintained by the Department of Health and the National Health Research Institutes of Taiwan, comprises comprehensive medical care information available for research purposes.

The NHIRD contains the medical records of approximately 23 million residents of Taiwan. The large sample size and longitudinal nature of the database provide advantages for nonexperimental studies, including observational and descriptive studies. The accuracy and validity of diagnoses in the NHIRD have been evaluated^28^. The database provides basic information on every individual insured by the NHI program, including patient characteristics, records of outpatient visits, hospital admissions, drug prescriptions, and disease status and management. The diagnostic codes used are formatted by the International Classification of Diseases, Ninth Revision, Clinical Modification (ICD-9-CM). The present study was approved by the Institutional Review Board of Taipei Medical University (approval number N201908055) and was conducted under the approved guidelines. Informed consent of the study patients was not required because the dataset consisted of de-identified secondary data released for research purposes.

### Study design and population

In this retrospective cohort study, we used the Registry for Catastrophic Illness Patients in the NHIRD to identify patients with Sjogren syndrome (SS) (ICD-9-CM 710.2). In Taiwan, rheumatologists can apply for a catastrophic illness card for any patient with SS who fulfills the criteria of the American–European Consensus Group for SS^29^. Applications for the card are scrutinized in a peer-review process. Also, we excluded patients with comorbidities such as systemic lupus erythematosus, rheumatoid arthritis, scleroderma, polymyositis, dermatomyositis, or hepatitis C to limit our study sample to those with pSS.

Patients with pSS were followed-up from the date of the initial diagnosis till the development of osteoporotic vertebral or hip fracture (ICD-9-CM 733.13, 733.14, 805, 820), death, or the end of study (Jan 1, 2000 to Dec 31, 2015). We excluded patients with pSS who (1) experienced ONJ and osteoporotic fracture before enrolment, (2) had pathological fractures (ICD-9-CM 733.1) or malignancies, or (3) did not use anti-osteoporotic drugs.

The non-pSS control cohort was randomly selected from 23 million NHI beneficiaries. All non-pSS patients were also followed-up until a diagnosis of osteoporotic fracture, whichever occurred first. The same exclusion criteria were applied to the non-pSS controls (Fig. 1).

### Exposure to antiosteoporotic drugs

Total supply in days and quantity of drugs was estimated from pharmacy claims originating from inpatient and outpatient settings and NHI-contracted pharmacies. Patients were classified into an alendronate (BP) group or calcitonin/raloxifene (non-BP) group according to their exposure during the follow-up. To ensure sufficient medication exposure, patients who received fewer than three prescriptions of the study drugs during the follow-up period were excluded. Because the usage of antiosteoporotic drugs appeared in different years during the study period and some patients changed their usage over time, we applied the usage of antiosteoporotic drugs as a time-varying covariate in the applied Cox model. The cumulative dose was determined by multiplying the number of pills dispensed by the prescribed dose and dividing this value by the recorded supply days. The dosage was presented as the defined daily dose (DDD), which has been established by the World Health Organization (WHO) as the average maintenance dose per day for a drug used for its main indication in adults.

### Identification of patients with osteonecrosis of the jaw

We first identified patients with ONJ with possible diagnosis codes (i.e. ICD-9-CM 73008, 73000, 73340, 73349, 73018, 73010, 73020, 73345, 73399, 52689, 7339, 5264, 5289, 5259, and 5269) proposed by Solomon et al.^30^.

### Exposure variables

In addition to pSS, demographic characteristics such as sex, age, and comorbidities were analyzed (Table 1). Pre-existing comorbidities related to osteoporosis, included diabetes mellitus (ICD-9-CM 220), hyperlipidemia (ICD-9-CM 272.0–272.4), hypertensive diseases (IDC-9-CM 401–405), rheumatoid arthritis (ICD-9-CM 714.0), ankylosing spondylitis (ICD-9-CM 720), thyroid disease (ICD-9-CM 243, 244, 242), esophagitis/esophageal ulcer (ICD-9-CM 530.1, 530.2), peptic ulcer disease (ICD-9-CM 531, 532, 533), anemia (ICD-9-CM 280, 281, 282, 283, 284, 285), and chronic kidney disease (ICD-9-CM 585), were also extracted.

### Statistical analysis

To reduce bias in comparing BRONJ between the pSS and comparison group, we employed a propensity score matching model to eliminate bias causing by the demographic characteristics of both groups and to make them comparable. Propensity scores were calculated using a logistics regression model with age, gender, and age at the initial use of antiosteoporosis drugs as variables. We employed a 1:4 matching for the pSS and comparison group for BP users and the same ratio for non-BP users. Besides, to evaluate the interaction effect of BP use and pSS, we applied 1:1:4:4 matching in this subgroup.

We analyzed data using the Cox proportional hazards model for comparing the risk of BRONJ between BP and non-BP users in the pSS cohorts (Table 2). Whether the dosage of BP increased the risk of BRONJ was identified using stratified analysis (Table 3) and interaction analysis (Table 5).

**Table 5:** Interaction between pSS and cDDD group in bisphosphonate and non-bisphosphonate users.

	Case 1: bisphosphonate users		Case 2: non-bisphosphonate users	
	ADJ. HR	95% CI	ADJ. HR	95% CI
pSS				
No (Ref.)	1.000		1.000	
Yes	2.509*	(1.141–5.515)	0.549	(0.145–2.076)
CDDD group				
< 224(Ref.)	1.000		1.000	
≥ 224	0.705	(0.385–1.292)	0.534	(0.262–1.089)
Interaction				
pSS*cDDD group	0.701	(0.254–1.932)	1.117	(0.160–7.782)

(1) *0.01 ≤ *p* < 0.05, **0.0001 ≤ *p* < 0.01, ***p < 0.0001. (2) ^‡^ADJ. HR was adjusted by sex, comorbidities, and age at the initiation of bisphosphonate use.

*pSS* primary Sjogren syndrome, *cDDD* cumulative dose with defined daily dose, *95% CI* 95% confidence interval, *ADJ. HR* adjusted hazard ratio, *Ref* reference.

Sensitivity analyses were conducted to identify the risk of BRONJ for patients with pSS in terms of cumulative dose of BPs and non-BP use. We calculated the cumulative dose with DDD (cDDD) as recommended by the WHO. The hazard ratio (HR) of patients with pSS was estimated relative to those without and analyzed the robust findings using the HR trend with different cDDD levels. SAS (version 9.4, SAS Institute, Cary, NC, USA) was used for all data analyses, and a *p* value of < 0.05 was considered statistically significant.
